# Worldwide Survey on Digital Assistive Technology (DAT) Provision

**DOI:** 10.1155/2024/9536020

**Published:** 2024-02-06

**Authors:** Isabel Margot-Cattin, Anne Deblock-Bellamy, Julie Wassmer, Ritchard Ledgerd, Claudia von Zweck, World Federation of Occupational Therapists (WFOT)

**Affiliations:** ^1^Occupational Therapy Department, School of Social Work and Health (HETSL), University of Applied Sciences of Western Switzerland (HES-SO), Lausanne 1010, Switzerland; ^2^World Federation of Occupational Therapists, Geneva, Switzerland

## Abstract

Occupational therapists have long been involved in assistive technology (AT) provision worldwide. AT is recognized by the World Health Organization (WHO) to enhance functioning, independence, and autonomy and ultimately promote well-being for people living with disabilities. With the digitalisation of societies, the everyday lives and occupations of individuals are changing, becoming more reliant on digital solutions. The development of digital assistive technology (DAT) also offers opportunities for people with disabilities to access, interact, and pilot the digital world. However, we do not know how occupational therapists are involved in DAT provision worldwide. A survey was conducted in the global occupational therapist's community in June 2022 to describe DAT provision and the factors influencing it. Occupational therapy practitioners were included (*n* = 660) in the analysis. In DAT provision, occupational therapists mostly provide advice to people, assess their needs, provide instruction or training, prescribe DAT, and fit DAT to people and their environment. The clients served through DAT provision are most frequently people with neurological impairments, chronic illnesses, sensory impairments, and older people. The reasons for providing DAT focus on education, work, school, and leisure. It is expected that DAT provision will enhance independence, self-esteem, occupational participation, and social relationships. Issues faced by occupational therapists when providing DAT are costs of product and funding schemes, sufficient knowledge, and access to knowledge sources. Survey respondents are mostly from Western countries with access to the Internet and the digital world, including having digital literacy, highlighting the digital divide that exists between world regions and countries, but also within countries worldwide. There is a need to continue research to better understand the issues related to digitalisation and the digital participation of people living with disabilities.

## 1. Introduction

Assistive technology (AT) is any product, equipment, and system either specifically designed and produced or generally available that enhances functioning, and independence, and ultimately promotes the well-being of people with disabilities in desired occupations [1, 2]. According to the World Health Organization (WHO), over 2.5 billion individuals require AT, a number expected to rise to over 3.5 billion by 2050 due to aging populations as well as the increased proportion of individuals living with chronic diseases [2].

To ensure that AT meets individuals' needs, the selection process involves several steps. Although AT delivery processes vary around the world, the majority of them can be identified through seven stages [3]: (1) initiation (first contact), (2) assessment of needs, (3) identification of the appropriate type of AT, (4) selection of the specific AT, (5) determination of funding, (6) implementation (delivering the equipment to the user, fitting, and training), and (7) management and follow-up (periodic verifications). Specialized expertise is necessary to guide individuals with disabilities to go through this process. Occupational therapists worldwide play a crucial role in providing assistive technology and advocating for its adoption [4–6]. Their comprehensive understanding of assistive technology linked with considering individual abilities, occupation, and environment, enhances accessibility, usability, and adoption of AT, through a thorough assessment of barriers and facilitators [7]. According to a recent worldwide survey conducted by the World Federation of Occupational Therapists (WFOT), occupational therapists are primarily involved in the early stages of the AT delivery process, including providing recommendations for AT selection, conducting suitability assessments, and providing training [8]. Occupational therapists mainly work with AT related to physical impairments such as those facilitating bathroom use (e.g., toilet chairs and handrails) or wheelchairs [9].

With the digitalisation of our society, the everyday lives and occupations of individuals are changing. As participation in an increasingly digitalised world requires competencies to navigate between the physical and digital world [10], occupational therapists are becoming involved in the provision of digital assistive technology (DAT) to support people with disabilities worldwide [11]. “Digital” serves as an umbrella term encompassing technology-based products. These digital technologies include electronic tools, systems, devices, and resources designed to generate, store, or process data [12]. DAT has the potential to enhance communication, increase independence, promote community participation, improve productivity, or improve the quality of life of individuals with disabilities. Many occupational therapists recognize the potential of DAT to improve the lives of individuals with disabilities [11]. Recent results show how DAT may positively impact the quality of life and independent living of individuals living with disabilities worldwide ([13]; Zager [14]), enhancing communication opportunities, increasing independence, promoting community participation, and improving productivity. Research has demonstrated the effectiveness of DAT across various age groups, particularly among children [15]. For children, DAT serves additional specific roles, such as educational, social skills training, and adaptive play [16], supporting occupational choices and opportunities.

The provision of DAT should be part of a comprehensive approach to intervention and may involve collaboration with several professionals such as occupational therapists, speech therapists, or technology specialists. Occupational therapists increasingly recognize the importance of digital literacy. Incorporating digital literacy instruction into their therapy plans [17] can enhance people's ability to participate in the community and improve their quality of life [18]. There are inequities in accessing digital literacy instructions and DAT. Certain nations exhibit a robust culture surrounding digital literacy, accompanied by ample resources and governmental support for digital literacy programs. Conversely, in other countries, the availability of digital literacy instruction may be constrained due to insufficient resources and funding. Still, occupational therapists' role in DAT provision is unclear. Factors influencing DAT provision, including DAT costs, access to funding for DAT, or needed expertise are also unclear. Thus, the present study is aimed at surveying how occupational therapists provide digital assistive technology (DAT), including the reasons for providing DAT, the issues they face, and the factors that influence them.

## 2. Materials and Methods

A cross-sectional descriptive study design was used. To explore the role of occupational therapy in the provision of digital assistive technologies, an online survey was created by the World Organization of Occupational Therapists (WFOT) in collaboration with Logitech SA. Logitech is a Swiss company focused on technological innovation that designs digital products like keyboards or trackballs to support people in connecting and interacting with the digital world. It sells products everywhere in the world and has been exploring the opportunity to expand into DAT product design. As such, Logitech was interested to learn more about the role of occupational therapists in the provision of DAT worldwide, as they were recognized as gatekeepers, as well as advisors in the design and assessment of DAT. Logitech approached WFOT to conduct this survey, which was in line with WFOT's previous surveys [8, 9].

The survey questionnaire was developed using a template that WFOT previously used for the AT survey in 2017. Many of the questions therefore had been previously used/piloted. A few specific questions were added based on a review of research evidence regarding the benefits of DAT or the reasons for using DAT, for example. The survey link was distributed by WFOT through social media and e-news publications to the global occupational therapy community and by email to WFOT member organizations. The survey was conducted between June and July 2022 (approximately 1 month) using SurveyMonkey software. The survey was translated by the WFOT Translation Teams composed of volunteer occupational therapists, to be available in French, German, and Spanish, as well as English. The translated versions of the survey were reviewed by a second translator to ensure accuracy. As both WFOT and Logitech are Swiss legal organizations and the analysis was done in Lausanne (Vaud), in Switzerland, the Vaud ethics committee (CER-VD) was consulted before the survey went online. A no-application request was submitted and approved (Req-2022-00367).

To be included in the study, participants had to identify themselves as occupational therapists, practice occupational therapy, and complete the survey. Managers, educators, and researchers were included only if they were also practicing occupational therapy. Before starting the survey, all participants were asked to give their consent to an anonymous use of their data for research and development purposes. An operational definition of DAT based on Wang et al. [12] was provided in the survey, as well as the indication of an incentive. The incentive was offered by Logitech, as each participant would add $5 to the WFOT fund for supporting occupational therapists from low-income countries to attend the WFOT Conference in Paris, in August 2022.

The survey consisted of 21 questions: 6 requested demographic information, 11 focused on DAT provision (e.g., the role of the occupational therapist, the target population, and the reasons for using DAT), 2 explored knowledge of the respondent regarding DAT, and 2 focused on quality of service and barriers to DAT provision. The survey collected quantitative data using closed-ended questions and multiple-choice answers or four-point rating scales to select their answer from “never” (1), “occasionally” (2), “often” (3), “very often” (4) or “low” (1), “medium” (2), “high” (3), “very high” (4). Responses were anonymous, and no personal information was collected. The first page of the online survey provided a briefing about the study, and consent was obtained from all participants before starting the survey.

Descriptive statistics were used to characterize participants, using ratios for the demographic questions of the survey. For questions rated on a 4-point scale, weighted averages were used to consider the importance or frequency of the data. Using weighted average scores offers a framework designed to facilitate understanding which of the answers is more important or more frequent [19], notably when trying to describe the practice in the health sector. Using a weighted average score was grounded in the need to highlight tendencies. All statistical tests were performed using the Statistical Package for Social Sciences (SPSS) computer software, version 27.

## 3. Results

1444 individuals answered the survey, of which 873 gave complete responses. Out of these, 660 fulfilled the inclusion criteria and were included in the analysis. 213 were excluded as they did not work with people receiving occupational therapy services. Thus, the respondents (*n* = 660) were occupational therapists, who worked with people receiving occupational therapy, including researchers, managers, and educators.

### 3.1. Characteristics of the Respondents

Together, the respondents represent over 70 countries, ranging from Europe to Africa and America to Asia; some countries only have a few respondents while others have more than 30. The most represented countries are France (82/12.4%), Canada (65/9.8%), United States (57/8.6%), Switzerland (38/5.8%), and Hong Kong (31/4.7%). Other countries have between 10 and 30 respondents: Singapore (29), Denmark (22), Argentina (23), Australia (20), Germany (18), Greece (18), United Kingdom (18), South Africa (18), Italy (14), Philippines (12), Kenya (12), and Taiwan (11).  Table 1 presents the age groups, years of experience, type of funding of workplace, and type of workplace.

### 3.2. Description of DAT Provision by Occupational Therapists

Provision of DAT by occupational therapists is mostly included as one aspect of interventions with their clients. Only 3.5% of respondents solely work on the provision of DAT. Occupational therapists refer to other occupational therapists for specific interventions regarding DAT provision (weighted average score = 2.2). Other rehabilitation team members (weighted average score = 2.0) also refer to occupational therapy for DAT provision. Family doctors or general physicians (GP) refer the least (weighted average score = 1.6). All client age groups are being served by DAT provision, with a slightly higher representation of adults (18-65 years old).  Table 2 describes the DAT provision by aims, health conditions of clients, focus, and reported benefits (see Table 2).

In addition, the respondents report a satisfaction rate of 82.9% from their clients and a self-efficiency rate of 80.5% on their DAT provision. Respondents use various sources for keeping up to date with knowledge about DAT provision (see Table 3). However, the main barrier to DAT provision reported is the cost of products. As such, most DAT provision happens when the product and services are free to the users.

Considering that the level of knowledge might influence the rating of both provision effectiveness and client satisfaction, as perceived by occupational therapists, Table 4 shows the frequencies and ratios for the respondents reporting enough or more than enough knowledge, compared to those indicating they had insufficient knowledge (see Table 4).

## 4. Discussion

Occupational therapists around the world play a role in the provision of digital assistive technology (DAT) to individuals with disabilities [20]. They are involved in all provision processes, although the design and production aspects are less represented in the results. Occupational therapists assess the needs, identify appropriate technology solutions, and provide training and support for the use of those technologies. They also identify treatment goals and objectives and use DAT to help achieve those goals and improve the quality of life of people with disabilities from various age groups [8].

Occupational therapists acknowledge the significance of digital literacy in the lives of their clients and are integrating digital literacy instruction into their therapy plans [17]. Digital literacy refers to using technology to find, evaluate, create, and communicate information [21]. It includes a range of skills such as the ability to use a computer, access the Internet, use email, and other digital tools [22]. Digital literacy also includes the ability to critically evaluate information found online, protect oneself from online threats such as scams and identity theft, and understand the ethical and legal implications of online behaviour. Digital literacy is becoming increasingly important in today's world as technology continues to play a larger role in all aspects of life, including maintaining jobs. Incorporating digital literacy instruction into therapy plans can greatly enhance people's ability to participate in the community and improve their quality of life [18]. However, there are inequities in accessing digital literacy instructions and DAT, which may partly be driven by cultural and economic factors [15]. In some countries, there is a strong culture of digital literacy and resources, as well as government funding for digital literacy programs, whereas, in other countries, the provision of digital literacy instruction may be limited by a lack of resources and funding. Therefore, the provision of DAT by occupational therapists can vary depending on the country and the resources available [23].

The results show that occupational therapists provide DAT for their clients for reasons that are in line with previous findings [24], like education, work or school, and leisure [16], especially with children [15]. Using digital games in occupational therapy practice is also a growing trend [25]. Digital games can provide an engaging and interactive way for clients to work on various therapeutic goals, such as improving fine motor skills, cognitive function, and social interaction [26]. It may also improve motivation through the “fun” component of those games and belonging as the person with disabilities feels more like everyone in their age group, having access to the same “games.”

Many respondents, predominantly from community-based services, cater to individuals, including older adults, with disabilities in community settings. The recognition of DAT supporting independent living at home is a key finding. Given the growing trend of aging in place and the expanding aging population, incorporating DAT provision and digital literacy instruction in community-based services is crucial [24]. Exploring the specific challenges and opportunities presented by DAT enriches our understanding and aids in developing tailored digital literacy programs for home care recipients Zager [14]. Addressing this aligns with evolving healthcare needs and may contribute to diminishing the impact of global occupational therapist shortages [11].

The survey respondents seem to indicate that they are less involved in the development of DAT, which is also in line with previous findings [27]. While collaborating with technology companies, occupational therapists may provide input on features, design, and usability of devices to ensure that the technology is accessible and user-friendly for clients with disabilities. Conducting research informs the effectiveness of DAT and provides feedback to developers on how to improve the technology, cross thresholds, meet expectations, and embrace possibilities, for example, for customizing existing technology or developing accessible/usable technologies [11, 28]. Occupational therapists may advocate and advise on guidelines and standards and provide training for other health professionals, clients, and caregivers on how to use DAT effectively. As the development of DAT often is a multidisciplinary effort, the inclusion of occupational therapists can help ensure that the technology is designed with the needs and abilities of clients in mind and that it is effective in achieving a better quality of life [29].

The cost of DAT can have a significant impact on its use. This was also reported by respondents in the study around the world. In many cases, DAT is expensive, and this can limit its availability and accessibility for individuals with disabilities, particularly those in low-income countries or communities, where access to healthcare and other services may be limited [23, 30]. Many governments and organizations do not have the funding to purchase DAT, which can limit its availability in schools, healthcare facilities, and other settings. Many insurance plans do not cover the cost of DAT, which can make it difficult for individuals to afford the technology they need. Maintenance and repair are also issues for individuals with poor access to healthcare. However, there are some initiatives and organizations that aim to reduce the cost of DAT and make it more accessible for individuals with disabilities and older adults [16, 31], like tailoring services to increase trust with technology adoption by older adults to use digital assistive technology to stay at home independently.

Although differences in the provision of DAT might not only be country-based but also affected by socioeconomic status, these elements were not indicated in the survey results when looking for factors influencing DAT provision (e.g., looking for country-specific or socioeconomic status). However, it is important to note that this is not only a problem in developing countries but it is also present in developed countries where socioeconomic status can directly affect access to DAT, maintenance of DAT, and technology in general [32, 33], creating a digital divide between individuals and population groups. A digital divide is recognized where there is an unequal distribution of access to technology and the Internet between different groups of people [33], which can affect DAT provision. As DAT often requires Internet access to function, individuals in areas with limited Internet access may not be able to use DAT as effectively. The digital divide can also vary within a country, by rural or urban areas, by gender, age, race, or ethnicity, among other factors, making it especially difficult to highlight specific factors influencing the provision of DAT in practice [33].

Knowledge about DAT is a limit reported by the survey respondents. Professionals who are not familiar with technology may have difficulty identifying appropriate DAT options for their clients or may not be able to effectively teach clients how to use the technology [34]. For occupational therapists specifically, there is a growing recognition that digital literacy is a core competency that should be included in the education and training of occupational therapists [17, 35, 36]. However, the lack of digital literacy training for occupational therapists may still be an issue in some countries or settings. When professionals lack digital literacy, it can also limit their ability to collaborate effectively with other professionals in the field, such as technology specialists and educators. This can make it more difficult to provide comprehensive and coordinated services about DAT to clients with disabilities. To address this issue, some occupational therapy education programs are starting to include more digital literacy training in their curriculum [36]. Additionally, professional development opportunities such as workshops, webinars, and online resources can be used to help professionals gain the necessary skills and knowledge. It is important for professionals to continuously update their critical digital literacy skills and knowledge and to collaborate with other professionals and organizations in the field of digital assistive technology to ensure they are up to date with the latest technology and best practices.

### 4.1. Study Limitations

The survey was distributed through the WFOT newsletter and WFOT network via digital support, targeting members. As such, the sampling of respondents is not representative of the occupational therapy practice community but rather represents those who are most involved in following the international development of the profession through WFOT. The survey findings may not be generalised to represent what is happening around the world but rather gives an idea of the trends and issues facing the profession in its development regarding DAT provision. It also offers ideas on the need for support in DAT provision, like increasing the offer of continuing education in the countries.

The survey also stayed online only for a little more than one month (June 2022) and was linked to the preparation of the WFOT Congress in August 2022 in Paris (France) due to the incentive included. Therefore, it is not surprising to find French respondents with the same number of respondents as the United States, although they count far fewer occupational therapists. However, respondents were mostly from Western countries, like the United States, Canada, Switzerland, Hong Kong, Australia, and Singapore, which have usually better Internet access and insurance covering for DAT provision. As countries from Africa or South America are not enough represented in the sample, the survey results do not inform on the situation faced in countries where the digital divide is prominent, calling for research targeting these issues.

## 5. Conclusions

In conclusion, this survey sheds light on the global landscape of occupational therapists' involvement in digital assistive technology (DAT) provision. The findings reveal that occupational therapists play a crucial role in advising, assessing needs, providing instruction, prescribing, and fitting DAT for individuals with neurological impairments, chronic illnesses, sensory impairments, and older adults. The focus on education, work, school, and leisure underscores the broad impact of DAT on various aspects of life. However, challenges such as cost, funding, and knowledge access indicate barriers to effective DAT provision. Moreover, the digital divide highlighted among respondents underscores the urgency of addressing global discrepancies in digital literacy and access. Continued research is essential to unravel and address these issues in the evolving landscape of digital participation for people with disabilities.

## Figures and Tables

**Table 1:** Most salient characteristics of respondents by ratio (%).

Age groups	40-49 (31.4%)	30-39 (25.7%)	20-29 (24.1%)		
Experience years	More than 10 (32.3%)	1-3 (22%)	Less than 1 (15.6%)		
Workplace funded	Publicly (37.4%)	Privately (19.7%)			
Type of workplace	Home care services (42.3%)	Schools and education (34.2%)	Rehabilitation centres (35.3%)	Community services (27.4%)	Hospitals (25.8%)

**Table 2:** Hierarchy of characteristics of DAT provision.

*Aims of DAT provision*	*Weighted average score*
Provide advice to people regarding DAT	2.6
Assess the needs of people for DAT	2.5
Provide instruction or training in the use of DAT	2.5
Prescribe or order DAT	2.3
Fit DAT to people and their environment	2.1
Refer to another health professional for DAT	2.1
Ensure follow-up, maintenance, and repairs	1.9
Designing, making, and building	1.5

*Health conditions of clients receiving DAT*	*Weighted average score*
Neurological conditions	2.7
Chronic diseases or illnesses	2.4
People with sensory impairments	2.3
Older people	2.1
People with mental health issues/illnesses	1.9
People with orthopaedic conditions	1.9
People with communicable diseases or illnesses	1.6
People with cardiopulmonary conditions	1.4

*Focus of DAT provision*	*Weighted average score*
Education	2.3
Work or School	2.3
Leisure	2.3
Device access	2.2
Communication	2.2
Mobility	2.0
Memory and organization	2.0
Environmental control	1.9
Activities of daily living	1.9
Social interactions	1.4

*Reported benefits of DAT provision*	*Weighted average score*
Independence	2.9
Self-esteem	2.7
Occupational participation	2.6
Social relationships	2.4
Safety	2.2
Caregiver burden reduction	2.2
Financial savings	1.5

**Table 3:** Additional factors for DAT provision.

*Reported knowledge level*			*Knowledge source*
Insufficient (37.3%) *N* = 246	Sufficient (44.2%) *N* = 292	More than sufficient (16.2%) *N* = 107	Internet searches (77.6%) Peer to peer demonstration (58.9%) Webinar or online courses (54.5%) Supplier recommendations (45.3%) Continuing education (41.2%)

*Identified barriers*			*Weighted average score*
Product costs			3.1
Suitable technology			2.6
Time or staff capacity to meet needs			2.6
Availability of suitable training			2.5
Availability of services			2.4
Follow-up and safety			2.4
Screening and referral			2.3
Travel distances			2.2

**Table 4:** Knowledge level (*n* = 645) indicated by effectiveness and satisfaction levels.

		Sufficient knowledge	Ratio	Insufficient knowledge	Ratio
	Total nb	399	100.00%	246	100.00%
Effectiveness	Low	31	7.77%	59	23.98%
	Medium	177	44.36%	127	51.63%
	High	139	34.84%	37	15.04%
	Very high	36	9.02%	6	2.44%
	Unknown + n/a	16	4.01%	17	6.91%

Satisfaction	Low	6	1.50%	13	5.28%
	Medium	113	28.32%	110	44.72%
	High	202	50.63%	74	30.08%
	Very high	50	12.53%	11	4.47%
	Unknown + n/a	28	7.02%	38	15.45%

## Data Availability

The survey data used to support the findings of this study are available from the corresponding author upon request.
